# Inducible expression of Wnt7b promotes bone formation in aged mice and enhances fracture healing

**DOI:** 10.1038/s41413-019-0081-8

**Published:** 2020-02-03

**Authors:** Deye Song, Guangxu He, Fangfang Song, Zhepeng Wang, Xiaochen Liu, Lele Liao, Jiangdong Ni, Matthew J. Silva, Fanxin Long

**Affiliations:** 1 Department of Orthopedics, The Second Xiangya Hospital, Central South University, Hunan, 410011 PR China; 2 Department of Orthopaedic Surgery, Washington University School of Medicine, St. Louis, MO 63110 USA; 3 Translational Research Program in Pediatric Orthopedics, The Children’s Hospital of Philadelphia, University of Pennsylvania, Philadelphia, PA 19104 USA

**Keywords:** Bone, Bone quality and biomechanics

## Abstract

There remain unmet clinical needs for safe and effective bone anabolic therapies to treat aging-related osteoporosis and to improve fracture healing in cases of nonunion or delayed union. Wnt signaling has emerged as a promising target pathway for developing novel bone anabolic drugs. Although neutralizing antibodies against the Wnt antagonist sclerostin have been tested, Wnt ligands themselves have not been fully explored as a potential therapy. Previous work has demonstrated Wnt7b as an endogenous ligand upregulated during osteoblast differentiation, and that Wnt7b overexpression potently stimulates bone accrual in the mouse. The earlier studies however did not address whether Wnt7b could promote bone formation when specifically applied to aged or fractured bones. Here we have developed a doxycycline-inducible strategy where Wnt7b is temporally induced in the bones of aged mice or during fracture healing. We report that forced expression of Wnt7b for 1 month starting at 15 months of age greatly stimulated trabecular and endosteal bone formation, resulting in a marked increase in bone mass. We further tested the effect of Wnt7b on bone healing in a murine closed femur fracture model. Induced expression of Wnt7b at the onset of fracture did not affect the initial cartilage formation but promoted mineralization of the subsequent bone callus. Thus, targeted delivery of Wnt7b to aged bones or fracture sites may be explored as a potential therapy.

## Introduction

The adult human skeleton is a dynamic organ that undergoes continuous remodeling throughout life to support whole-body mineral homeostasis while sustaining structural integrity. Healthy remodeling requires the balancing activities of bone resorption by osteoclasts and bone formation by osteoblasts.^1^ Loss of the balance in favor of bone resorption causes a net bone loss, resulting in osteopenia or in the more severe cases osteoporosis, a hallmark of aging. The current therapeutics for osteoporosis include two bone anabolic agents, namely teriparatide and abaloparatide, both recombinant proteins that activate the parathyroid hormone receptor PTH1R.^2^ However, the clinical use of those drugs is limited due to unresolved concerns about osteosarcoma.^3^ Thus, additional bone anabolic therapies are necessary to fulfill the unmet needs among osteoporotic patients. Such new therapies are also needed to promote bone fracture healing which is often negatively impacted by aging and diabetes.^4,5^

Wnt signaling has emerged as a promising target pathway for developing new bone anabolic drugs.^6,7^ Wnt proteins are a family of secreted glycoproteins that signal through cell-surface receptors including the frizzled proteins and the low-density lipoprotein receptor-related protein Lrp5 or Lrp6.^8^ Wnt signaling is fine-tuned by various extracellular modulators, including the secreted antagonist sclerostin (encoded by the Sost gene), that compete with Wnt proteins for binding to Lrp5/6.^9^ Genetic evidence has demonstrated that inhibitory or activating mutations of LRP5 in humans cause osteoporosis-pseudoglioma (OPPG) or high-bone-mass syndromes, respectively, whereas deletion or loss of expression of Sost results in osteosclerosis.^10–14^ In addition, deficiency of WNT1 has been linked to inherited early-onset osteoporosis or osteogenesis imperfecta in patients,^15–18^ whereas WNT16 has been repeatedly associated in genome-wide association studies with bone mineral density (BMD), cortical bone thickness, and nonvertebral fractures.^19,20^ Those findings thus establish Wnt signaling as an essential bone-promoting mechanism and raise the potential that the pathway may be targeted for developing bone anabolic drugs. Indeed, a humanized monoclonal antibody against sclerostin, known as Romosozumab (ROMO), has recently been approved for clinical use. Potential pharmaceutical use of Wnt proteins per se however has not been fully explored.

Wnt7b has been implicated in the stimulation of bone formation. In the developing long bones of the mouse, Wnt7b is enriched in the osteogenic perichondrium (PC) flanking the hypertrophic cartilage but declines in the more mature osteoblasts, indicating transient upregulation of Wnt7b during osteoblast differentiation in vivo.^21,22^ Wnt7b has also been shown to increase with osteoblast differentiation of calvarial cells in vitro.^23^ Functionally, Wnt7b deletion in the osteogenic progenitors of the mouse causes a temporary delay in ossification but the defect is later resolved perhaps due to compensation by the other Wnt proteins.^21^ In adult bone, Wnt7b expression appears to be minimal but it is induced by mechanical loading.^24^ Importantly, the extent of Wnt7b induction declines with aging and may underlie the muted bone anabolic response to mechanical loading in the aged mice.^24^ Aging also reduces WNT7B expression in human bone marrow (BM) stromal cells, indicating that the decline of Wnt7b expression in osteoblast lineage cells may contribute to the diminution of bone formation activity associated with aging.^25^ These findings raise the interesting possibility that supplying additional Wnt7b may help to rejuvenate aged bone. We have previously reported that overexpression of Wnt7b in the osteoblast lineage potently stimulates bone formation in adult mice by increasing both osteoblast number and activity.^26^ That work however relied on sustained expression of Wnt7b beginning in the embryo and could not discern a potential bone anabolic effect specifically in the aged bone.

Here we report that temporal induction of Wnt7b expression in the aged mouse bones exerts a potent bone anabolic function. Furthermore, postfracture application of Wnt7b notably stimulates mineralization of the bone callus. These results therefore provide proof of principle that Wnt7b protein may be explored as a bone anabolic therapy.

## Results

### Generation of the Osx-rtTA mouse

**Fig. 1:**
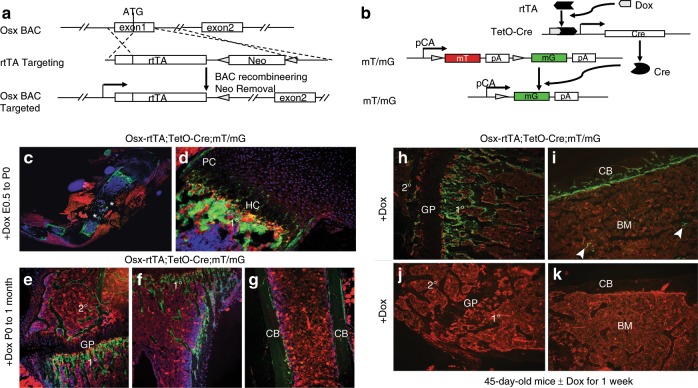
Generation and validation of the Osx-rtTA transgenic mouse. **a** Generation of Osx-rtTA. **b** Validation of the transgenic mouse by crossing with tetO-Cre and mT/mG mice. In **b**, the rtTA protein expressed by the Osx-rtTA transgene, upon binding to doxycycline (Dox) supplied through the diet, activate expression of Cre, which in turn recombine the mT/mG allele to convert expression of membrane-tethered tdTomato (mT) to membrane-tethered eGFP (mG). **c**, **d** Direct visualization of GFP and RFP on sections of the forelimb from Osx-rtTA;tetO-Cre;mT/mG mice receiving Dox from conception to harvest at birth. Note GFP specifically in metaphysis and diaphysis of the humerus (**c**). Asterisk indicates artifactual detachment of tissue. At higher magnification (**d**), GFP was detected in the perichondrium (PC), the hypertrophy cartilage (HC), and the primary spongiosa (1°). **e**–**g** Sections of the tibia from Osx-rtTA;tetO-Cre;mT/mG mice receiving Dox from birth to 1 month of age when the mice were harvested. Note GFP on bone surfaces of primary (1°), secondary (2°) ossification centers, and the cortical bone (CB), but not in the growth plate (GP). Sections of the tibia from Osx-rtTA;tetO-Cre;mT/mG mice with (**h**, **i**) or without (**j**, **k**) Dox from 45 days of age for 1 week before harvest. Note that the bone marrow proper (BM) was largely negative for GFP except for the occasional stromal cells (arrowheads) in the +Dox mouse (**i**).

To create a mouse genetic tool that allows for temporal control of gene expression in the osteoblast lineage, we set out to create a transgenic mouse (termed Osx-rtTA) expressing the reverse tetracycline transactivator (rtTA) from the Osx locus. Briefly, an Osx BAC (bacterial artificial chromosome) was modified to replace the first exon of Osx with the cDNA for rtTA, and then used to produce transgenic mice through pronuclear injection (Fig. 1a). The founder animals carrying the Osx-rtTA transgene were mated with the tetO-Cre and the mT/mG mice to produce triple transgenic animals with the genotype of Osx-rtTA;tetO-Cre;mT/mG, which were tested for green fluorescence protein (GFP) expression in response to doxycycline (Dox) added to either the food or the drinking water (Fig. 1b). Two founders were found to produce triple transgenic progenies that express Cre activity in skeletal tissues only in the presence of Dox. As the two founders exhibited similar expression patterns, we present here the data from only one of them (line 4) and propagated this line for subsequent studies. When the Osx-rtTA;tetO-Cre;mT/mG embryos were exposed to Dox throughout embryogenesis and analyzed at birth, GFP was readily detected in the bone shaft of long bones (Fig. 1c). At a higher magnification, GFP was notable within the primary spongiosa (1°) and at the PC flanking the hypertrophic zone, both of which are known to undergo active osteoblastogenesis at this stage (Fig. 1d). GFP was also detectable in the hypertrophic chondrocytes (HC), indicating that the BAC transgene recapitulated the normal expression pattern of Osx in skeletal cells, including that in the embryonic HC (Fig. 1d).^27^ No GFP was detected in tendons, ligaments, skeletal muscle, or marrow fat. Outside the skeleton, GFP was not detected in the brain, intestine, heart, lung, or liver either in whole-mount or on sections. When the triple transgenic mice were exposed to Dox from birth to 1 month of age, GFP was found to decorate all bone surfaces in both the primary (1°) and secondary (2°) ossification centers, as well as those of the cortical bone (CB) (Fig. 1e–g). However, the growth plate (GP) including the hypertrophic zone no longer expressed GFP when Dox was applied postnatally (Fig. 1e). When the triple transgenic mice were treated with Dox for 1 week starting at 45 days of age, GFP was also largely restricted to the primary (1°) and secondary (2°) ossification centers together with the CB, with the exception of occasional signals in some stromal cells of the BM (Fig. 1h, i). The activity was strictly dependent on Dox as triple transgenic mice without Dox exposure expressed no GFP in the skeleton (Fig. 1j, k). Thus, the Osx-rtTA transgenic mouse can be used for temporal manipulation of gene expression in the osteoblast lineage through the use of Dox.

### Expression of Wnt7b in aged bones increases bone mass

**Fig. 2:**
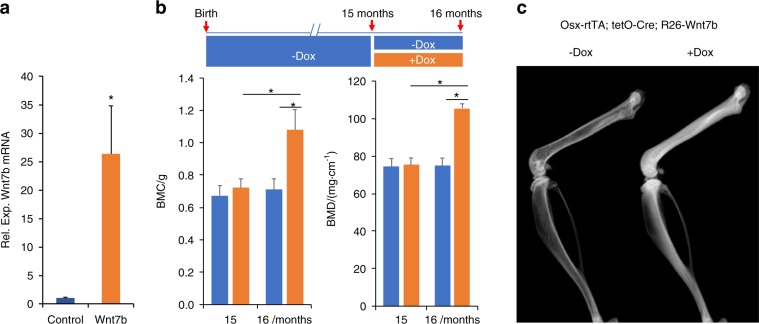
Activation of Wnt7b in aged mice increases bone mass. **a** RT-qPCR analyses of Wnt7b mRNA in the bones of mice treated with Dox for 2 weeks starting at 4 weeks of age. Ctrl: Osx-rtTA;R26-Wnt7b mice; Wnt7b: Osx-rtTA;tetO-Cre;R26-Wnt7b mice. *P < 0.05, *n* = 3. **b** Whole-body (excluding head) DXA analyses of bone mineral content (BMC) and bone mineral density (BMD) of Osx-rtTA;tetO-Cre;R26-Wnt7b mice with or without Dox treatment from 15 through 16 months of age. *P < 0.05, one-way ANOVA, *n* = 5 (four males, one female). **c** Contact X-ray images representative of the hindlimbs at time of sacrifice. Error bars: stdev in all figures.

**Fig. 3:**
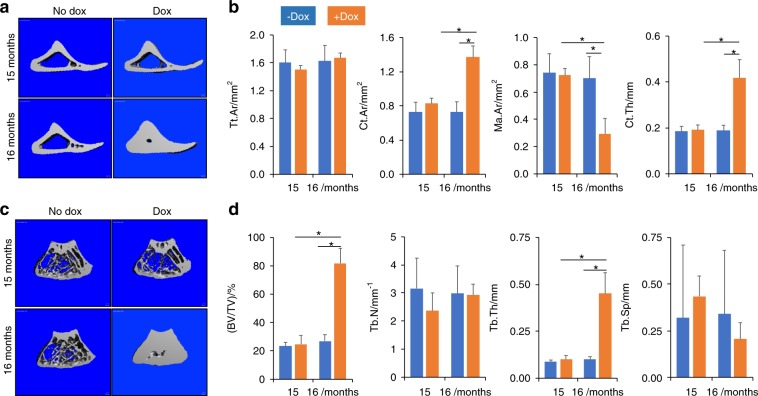
VivaCT analyses of mouse bones. **a** 3D reconstruction images of the tibial cortical bone at the tibiofibular junction. **b** Quantification of the cortical bone parameters in the tibia. **c** 3D reconstruction images of the metaphyseal region of distal femur. **d** Quantification of the trabecular bone parameters in the femur. *P < 0.05, one-way ANOVA, *n* = 5 (four males, one female).

To confirm the efficacy of Osx-rtTA in activating Wnt7b expression in bone, we mated the Osx-rtTA mouse with the tetO-Cre and the R26-Wnt7b mice to generate triple transgenic progenies with the genotype of Osx-rtTA;tetO-Cre;R26-Wnt7b, with one copy of each transgene. The triple transgenic mice, together with Osx-rtTA;R26-Wnt7b littermate control, were subjected to Dox treatment for 2 weeks beginning at 4 weeks of age before RNA was harvested from the long bones. RT-qPCR showed that Wnt7b was overexpressed by ~26-fold in the triple heterozygous over the control mice, indicating effective activation of the R26-Wnt7b allele by Osx-rtTA in bone (Fig. 2a). We next set out to test the potential effect of Wnt7b on aged bones. The triple transgenic mice were raised until 15 months of age with regular chow before being randomly divided into two groups with or without Dox added to the food for 1 month before sacrifice (Fig. 2b). The bone parameters were collected with dual energy X-ray absorptiometry scan (DXA) and in vivo micro-computed tomography (vivaCT) immediately before and after the Dox regimen. DXA indicated that Dox markedly increased both bone mineral content and BMD in the mice after 1 month of treatment, whereas those parameters remained constant in the control mice (−Dox) during the same period (Fig. 2b). Contact X-ray radiography confirmed that the bones of animals receiving Dox (+Dox) exhibited much greater density than those without Dox (−Dox) (Fig. 2c). 3D reconstruction of the CB from vivaCT scans of the tibia demonstrated that Dox caused a marked thickening of the cortex, resulting in virtual occlusion of the marrow space (Fig. 3a). Quantification of the CT data revealed that, whereas the overall size of the tibia (Tt.Ar) was not changed, the CB area (Ct.Ar) and thickness (Ct.Th) were significantly increased, thus greatly diminishing the BM space (Ma.Ar) in the +Dox group (Fig. 3b). Similarly, the trabecular bone of the distal femur was notably increased by Dox, as evident in the 3D reconstruction images (Fig. 3c). Quantitative analyses indicated that the increase in bone mass (BV/TV) was largely caused by thickening of the bone trabeculae in response to Dox (Fig. 3d). Thus, induction of Wnt7b expression in the aged bones for 1 month notably increases both cortical and trabecular bone mass.

### Wnt7b stimulates bone formation in aged mice

**Fig. 4:**
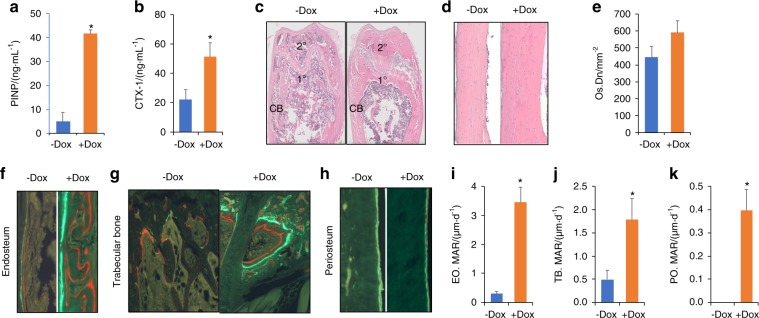
Wnt7b stimulates bone formation. Serum biochemical assays for bone formation marker P1NP (**a**) and resorption marker CTX-1 (**b**). H&E images of distal metaphysis (**c**) or diaphyseal cortical bone of the femur (**d**). 1°: primary ossification center; 2°: secondary ossification center; CB: cortical bone. **e** Average osteocyte density in cortical bone. **f**–**h** Representative images of calcein–alizarin red double labeling in the tibia. Quantification of Mineral Apposition Rate (MAR) at the endosteal (**i**), trabecular (**j**), or periosteal (**k**) bone surface. *P < 0.05, Student’s *t* test, *n* = 3 males. Females showed similar results.

To determine the cellular basis for the increased bone mass by Wnt7b, we performed biochemical assays with sera collected from the mice at the time of sacrifice. The bone formation marker P1NP (procollagen type I amino-terminal propeptide) was increased by an average of eightfold, whereas the bone resorption marker CTX-1 (carboxyl-terminal telopeptide of collagen type I) was also elevated by ~2.5-fold by Dox (Fig. 4a, b). Thus, bone formation was clearly activated relative to resorption and thus responsible for the overall increase in bone mass in response to Wnt7b.

We next performed histomorphometry for a better understanding of the bone phenotype. Hematoxylin and eosin (H&E) staining of femoral sections confirmed the presence of more cancellous bone in both primary (1°) and secondary (2°) ossification centers, and also thicker CB in the +Dox mice (Fig. 4c, d). A closer examination of the CB indicated that Dox caused a trend of increase in osteocyte density even though the change did not reach statistical significance (*n* = 3, *P* = 0.054) (Fig. 4e). To monitor the bone forming activity directly, we conducted double labeling experiments before the harvest of the animals. Images of the sections indicated a notable increase in the extent and intensity of fluorescence labeling on endosteal, trabecular, and periosteal bone surfaces (Fig. 4f–h). Quantification of the labeled surfaces confirmed that mineral apposition rate was markedly increased on all three bone surfaces, even though our earlier vivaCT analyses did not detect a statistically significant increase in periosteal growth (Fig. 4i–k). Overall, Wnt7b exhibits a potent bone forming activity in both trabecular and CB compartments of aged mice.

### Wnt7b enhances bone healing

**Fig. 5:**
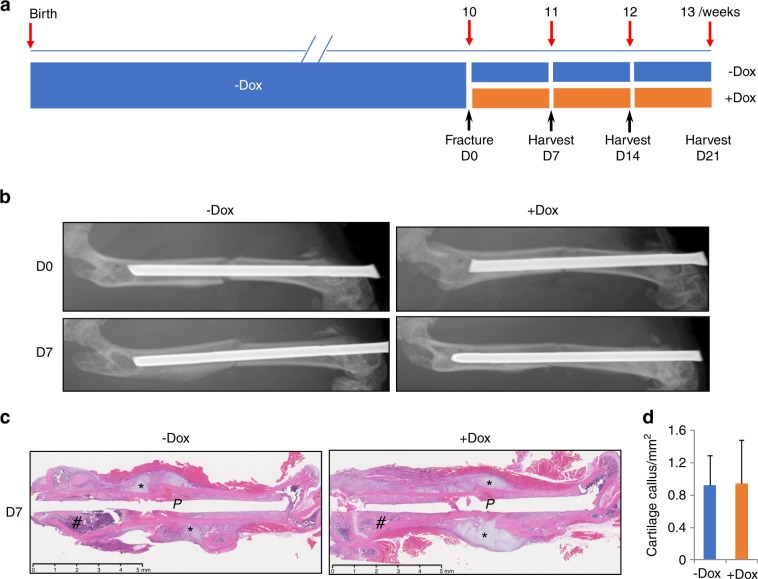
Wnt7b overexpression does not affect the cartilage callus of fractures. **a** A schematic of experimental design. **b** Representative X-ray images of femurs immediately following fracture (D0) and at 7 days after procedure (D7). **c** Representative H&E images of medial sections through the fractured femur at D7. *P* stabilizing pin, asterisk indicates cartilage callus. Note excessive bone accrual in distal metaphyseal region (#) of the +Dox mouse. **d** Measurements of cartilage callus areas on sections. *N* = 3 mice. Three sections per mouse were measured with the NanoZoomer NDP software.

We next sought to examine the effect of Wnt7b on bone healing in a closed femoral fracture model. Briefly, mice with the genotype of Osx-rtTA;tetO-Cre;R26-Wnt7b were raised on a regular diet until 10 weeks of age when a fracture was introduced at the diaphysis of the right femur of each mouse; the mice were then randomly assigned to two groups with or without Dox supplement in the diet, and allowed to heal for up to 21 days (Fig. 5a). Contact X-ray radiography immediately following the procedure confirmed a complete fracture at the middle point of the femur and the proper stabilization by a metal pin (Fig. 5b, upper). After 7 days of healing when an unmineralized cartilage callus normally forms, no “bridging” of the fracture was detected by X-ray regardless of the Dox treatment (Fig. 5b, lower). Histology of the femoral sections showed an obvious cartilage callus surrounding the fracture site regardless of Dox (Fig. 5c). Measurements of the cartilage callus area on sections indicated no difference in the callus size between the groups (Fig. 5d). No bony covering or encroachment of the cartilage callus was evident in the Dox-treated mice. However, excessive bone formation was noted in the distal medullary space next to the metal pin in response to Dox, indicating that Wnt7b was induced and functional in the femur at this 7-day time point (Fig. 5c). Thus, application of Wnt7b at the onset of fracture healing does not affect the early healing responses including cartilage callus formation.

**Fig. 6:**
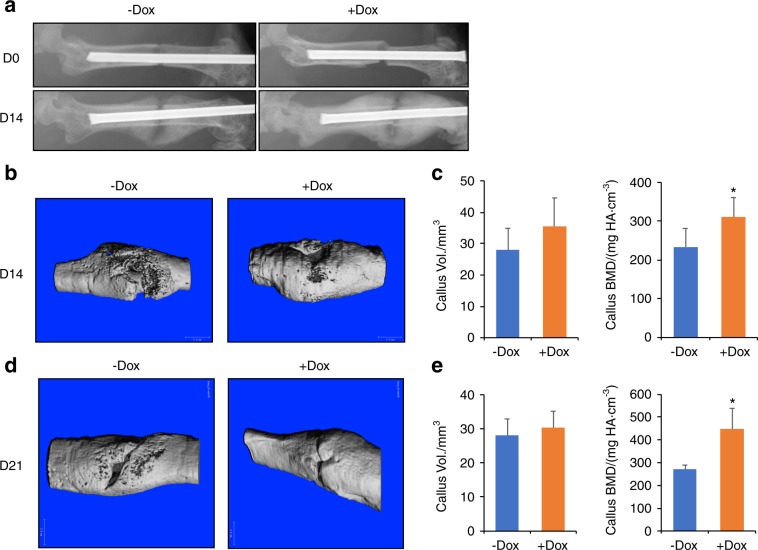
Wnt7b enhances mineralization of the bone callus. **a** Representative X-ray radiographs of femurs immediately following fracture (D0) and at 14 days afterwards (D14). Representative 3D reconstruction images (**b**) and quantification from microCT scans (**c**) of bone calluses at D14. *P < 0.05, Student’s *t* test, *n* = 7 or 6 for −Dox or +Dox groups, respectively. Reconstruction images (**d**) and quantification from microCT scans (**e**) of bone calluses at D21. *P < 0.05, Student’s *t* test, *n* = 3.

To assess the potential effects of Wnt7b on the later stages of fracture healing, we examined the bones at day 14 or 21 after fracture. At day 14 (D14), the fracture site normally contains a large osteochondral callus, with cartilage tissue starting to be replaced by bone, whereas day 21 (D21) is nearing completion of the cartilage-to-bone transition. At D14, X-ray radiography detected a marked increase in the radiodensity of the bony callus along with the rest the femur in the Dox-treated mice (Fig. 6a). MicroCT imaging of the fracture site confirmed the presence of a more contiguous bony callus across the fracture site in the +Dox mice (Fig. 6b). Quantification of the microCT data showed that although the overall callus volume did not significantly differ between the groups, the callus BMD was higher in the Dox group, indicating relatively more mineralized bone in the callus, a measure for accelerated healing (Fig. 6c). Similarly, at D21, microCT imaging showed that the bone bridging across the fracture site was more complete in the Dox-treated animals (Fig. 6d). The callus BMD became markedly higher in the +Dox group than the control, even though the overall callus size remained the same (Fig. 6e). Overall, Wnt7b stimulates mineralization of the bone callus with no obvious effect on the callus size.

## Discussion

We have developed a doxycycline-inducible system to manipulate gene expression in the osteoblast lineage of the mouse. By temporally activating Wnt7b expression in aged mice, we show that Wnt7b potently stimulates bone formation and leads to a marked increase in bone mass. We further demonstrate that Wnt7b promotes bone mineralization during fracture healing. The present work therefore provides evidence that targeted delivery of Wnt7b may be a potential therapy for osteoporosis and recalcitrant fractures.

Studies of gene function at specific embryonic or postnatal stages require genetic tools that can be controlled temporally. Previously, the Osx-Cre mouse strain was designed to express Cre in the osteoblast lineage in the absence of Dox.^28^ With that model, temporal gene manipulation specifically in postnatal mice requires long-term exposure of mice to Dox that begins in utero and extends till the stage when Cre activity is desired, as exemplified by a previous study.^29^ Long-term usage of Dox can be cumbersome, and more importantly may directly impact the skeleton.^30^ In addition, Osx-Cre may need extended “washout” time following the withdrawal of Dox in order for Cre to be expressed due to the high affinity of Dox for bone minerals.^31^ The Osx-rtTA transgene created here operates by the opposite design, where rtTA is expressed in the osteoblast lineage cells but becomes active only when Dox is provided. The Osx-rtTA transgene offers the flexibility of combining with either tetO-Cre for Cre/loxP-based genomic deletion, or other tetO-driven transgenes for direct overexpression of the desired gene. The new system therefore offers a useful tool for inducible genetic studies in bone.

The present study provides further evidence for the potent bone anabolic activity of Wnt7b. Although previous work has documented the strong osteogenic activity of Wnt7b when it is continuously overexpressed from embryogenesis onward, the current results provide the first evidence that Wnt7b application in aged bones markedly stimulates bone formation.^22,26^ The bone forming activity was most robust in the trabecular and endosteal bone surfaces, resulting in a notable increase in both cancellous and CB mass. Wnt7b also activated bone formation on the periosteal surface, but did not cause a statistically significant increase in the overall size of the long bones according to vivaCT, perhaps due to the relatively short duration of Wnt7b expression and the insufficient power of the sample size. In any case, the anabolic response to Wnt7b at the periosteum is clearly lesser than that at the trabecular or endosteal bone surface, and is in keeping with our previous observation in mice with continuous expression of Wnt7b in bone up to 2 months of age.^26^ Similarly, transgenic mice overexpressing Wnt16 in osteoblasts in two independent studies also did not increase the outer circumference of the bone shaft in long bones although one study showed an increase in endosteal bone growth.^32,33^ On the other hand, the injection of a sclerostin monoclonal antibody in 4-month-old mice for 5 weeks was shown to increase the overall bone size.^34^ Thus, Wnt proteins other than Wnt7b and Wnt16 may preferentially stimulate periosteal growth under normal conditions. Mice overexpressing Wnt10 or Wnt1 in bone have also been shown to increase bone mass but the effect on periosteal growth was not quantified in those studies.^35,36^ Our Wnt7b overexpression model described here is most comparable to that of Wnt1, as both proteins are expressed from a Rosa26 knockin allele (R26-Wnt7b or R26-Wnt1, respectively) upon Cre recombination. Activation of R26-Wnt7b or R26-Wnt1 in bone dramatically increases bone mass mainly through activation of trabecular and endosteal bone formation, and in both case mTORC1 signaling partially mediates the anabolic function.^26,35^ In both models, the overall bone resorption activity as indicated by the serum CTX-1 levels is increased over the control, even though the osteoclast number normalized to bone surface is reduced in the Wnt7b overexpression model.^26^ In contrast, serum CTX-1 levels were suppressed by sclerostin antibodies in both mice and in humans, indicating that Wnt proteins other than Wnt1 and Wnt7b play a more prominent role in suppressing bone resorption.^34,37^ Wnt16 is a potential contributor in this regard as Wnt16 deletion led to increased bone resorption in the mouse although mice overexpressing Wnt16 in osteoblasts exhibited normal serum CTX-1 levels.^32,38^ A limitation of the R26-Wnt1 or the R26-Wnt7b mouse model is that once activated by Cre, the expression of the Wnt protein is permanent. Thus, a future reversible model would be necessary in order to determine the state of bone modeling after the cessation of Wnt treatment. At least two other Wnt proteins, namely Wnt5a and Wnt4, were overexpressed in bone from the Rosa26 locus, but neither was reported to increase bone mass in the mouse.^26,39^ Thus, Wnt7b and Wnt1 are among the most potent bone-inducing Wnt proteins known to date.

Our fracture studies show that Wnt7b increases the mineral density of the bone callus without affecting the initial size of the cartilage callus. We did not detect a statistically significant increase in the total callus size by microCT at either D14 or D21. Thus, Wnt7b appears to mainly enhance formation and/or mineralization of the bone matrix, but future double labeling experiments would be necessary to determine whether Wnt7b increases bone formation rate in the callus. Future mechanical testing studies are also needed to determine whether Wnt7b improves the fracture site strength at the later time points. The fact that Wnt7b did not affect the formation of cartilage callus is worth noting. As Wnt/β-catenin signaling is known to inhibit chondrogenesis, the result is consistent with previous findings that Wnt7b signals largely through β-catenin-independent pathways.^21,26,40^ Different from Wnt7b, PTH has been shown to increase cartilage callus formation in fracture studies.^41^ Thus, in clinical settings, Wnt7b may be most useful in cases where the initial healing is relatively normal but the subsequent ossification is impaired or delayed.

Although ROMO, a humanized monoclonal antibody against sclerostin, was recently approved for osteoporosis treatment, it carries a black box warning against increased risk of cardiovascular events.^42^ Considering ROMO likely increases signaling by multiple Wnt proteins which may have nonoverlapping functions, targeting specific Wnt ligands such as Wnt7b may afford an opportunity to achieve the bone anabolic function while avoiding the unwanted side effects.

## Materials and methods

### Mouse strains

All procedures with mice were approved by the Animal Studies Committee at Washington University in St. Louis School of Medicine, or the IACUC committee at the Children’s Hospital of Philadelphia. The tetO-Cre mice and the mT/mG mice were purchased from the Jackson Laboratories.^43,44^ The R26-Wnt7b mouse strain was generated as previously described.^26^ Both males and females were included in the study.

To generate the *Osx-rtTA* transgene, we modified a *Osx* BAC (bacterial artificial chromosome, clone# RP23-399N14) (Children’s Hospital of Oakland Research Institute) to replace the first exon of *Osx* with the cDNA for rtTA2^S^-M2.^45^ Briefly, a 491 bp PCR amplicon immediately upstream of the *Osx* starting ATG (forward primer: 5′ CTACCCAGGTACAGACACT GGGCAGTTCTG3′; reverse primer: 5′ CCTCGAGCTGGGGACCGGGTCCCAAGGAGT3′), the cDNA for rtTA2^S^-M2 excised from pUHrT62-1,^45^ and a 367 bp PCR amplicon located ~100 bp downstream of the *Osx* starting ATG (forward primer: 5′ ATCCTCACATCGACA GGAGCTGCAATGCTAGTC3′; reverse primer: 5′ CTCTTTCCTCAACACAGACCTGACCAGATC 3′) were inserted into the pSV-Flp vector. The resulted plasmid was digested with restriction enzymes to release the targeting construct. Subsequent BAC recombineering was performed as described.^46–48^ Correctly targeted BACs were confirmed by directional PCR, restriction digestion, and sequencing. The finished *Osx-rtTA* BAC transgene is prepared for microinjection by modified Qiagen maxi-prep followed by dot dialysis of the circular BAC. Transgenic mice were produced by pronucleus injection at the Washington University Mouse Genetics Core. Seven founders were produced and were individually crossed with tetO-Cre and mT/mG mice to produce triple transgenic mice with or without doxycycline-containing drinking water (500 μg·mL^−1^, 2% sucrose) or food (200 mg·kg^−1^ food, Bioserv S3888). Bones and various internal organs were fixed with 4% paraformaldehyde overnight at 4 °C and subjected to cryostat sectioning. The adult bones were decalcified with 14% EDTA on a shaker for 3 days with daily change of solutions, and sectioned with the aid of Cryojane. GFP or RFP was directly visualized under a fluorescence microscope (NIKON ECLIPSE E800) equipped with a QImaging Retiga 2000R CCD camera. Founder mice that produced progenies expressing GFP in the absence of Dox or failed to express GFP specifically in skeleton were eliminated. Two founders exhibited the desired inducibility and specificity, and also stably transmitted the transgene through germline. Line 4 was chosen for further studies.

### RT-qPCR

Femurs and tibias were harvested from Osx-rtTA;tetO-Cre;R26-Wnt7b or Osx-rtTA; R26-Wnt7b littermate mice fed with Dox food for 2 weeks starting at 4 weeks of age. The bone shafts were collected in 1 mL Trizol (Thermo Fisher Scientific) after the GPs were surgically removed and the BM discarded by centrifugation. The bone shafts were homogenized with Precellys Evolution homogenizer (Bertin Instruments) and then extracted for RNA with the QIAGEN RNeasy Kit (#74104). RT-qPCR was performed with SuperScript™ VILO™ cDNA Synthesis Kit (11754050), followed by Power SYBR™ Green RNA-to-CT™ 1-Step Kit in a QuantStudio3 machine (Applied Biosystems). β-actin was used as internal control. The PCR primers are as follows: Wnt7b: 5′caatggtggtctggtacccaa3′, 5′agtctcatggtccctttgtggtt3′; β-actin: ′GTGACGTTGACATCCGTAAAGA3′, 5′GCCGGACTCATCGTACTCC3′.

### Bone morphometric studies

To monitor bone accretion with time, in vivo microCT (vivaCT 40; Scanco Medical) was used to scan the femurs and tibia in live mice, with the settings of 21 μm voxel size, 55 kVp, 145 μA, and 300 ms integration time. For quantifying trabecular bone parameters, 30 slices (0.6 mm total) immediately proximal to the distal femoral GP were analyzed (threshold set at 160). For quantifying CB parameters, 50 slices (1.0 mm total) starting at 5 mm above the tibia–fibular junction were analyzed (threshold set at 250). DXA scans and X-ray radiography were performed with Faxitran Model UltraFocus 100 (Tucson, AZ).

For histology, bones were fixed in 10% buffered formalin overnight, and then decalcified in 14% EDTA for 2 weeks. The decalcified bones were processed, embedded in paraffin and then sectioned at 6 μm thickness for H&E staining. For double labeling, calcein and alizarin were injected intraperitoneally at 20 mg·kg^−1^ at day 7 and 2, respectively, before the mice were sacrificed (Sigma, St. Louis, MO, USA). The bones were then fixed in 70% ethanol and processed for sectioning in methyl-methacrylate. Quantification of the histomorphometric parameters was performed with a Bioquant software (Nashville, TN, USA).

### Serum CTX-I and P1NP assays

For serum cross-linked C-telopeptide (CTX-I) and amino-terminal propeptide of type I procollagen (P1NP) assays, serum was collected from mice after 6 h of fasting. Assays were performed with RatLaps ELISA or Rat/Mouse P1NP EIA kit (both from Immunodiagnostic Systems, Ltd.).

### Bone fracture studies

Bone healing studies were performed with 10-week-old mice by using a closed femoral fracture model as previously described.^49^ Briefly, anesthetized mice were prepared for surgery by shaving the fur around the knee area and disinfecting the skin with Betadine. An incision was made at the right knee to dislocate the patella and the intramedullary canal was accessed by creating a hole in the distal end of the femur. A guidewire was placed into the femur and the mouse secured into custom three-point bending fixtures on a materials-testing machine (Dynamight 8841, Instron). The femur was fractured at the mid-diaphysis by application of a controlled transverse displacement. Then the femur was stabilized with a 24-gauge stainless steel pin that was positioned over the guidewire. The guidewire was removed, the rod trimmed and the wound sutured closed. Animals were given Buprenex to alleviate any pain. X-ray radiographs were taken with 20 s exposures at 25 kV immediately following surgery and prior to sacrifice to confirm pin stability (Faxitran UltraFocus 100, Tucson, AZ). If the radiograph showed displacement of the pin, the femur was excluded from all analyses.

The fractured femurs were analyzed at 7, 14, or 21 days after surgery. The femurs were fixed in 10% neutral buffered formalin at room temperature overnight, rinsed with PBS, and preserved in 70% ethanol until use. For CT scans, the femurs were embedded in 2% agarose and scanned with the settings of 21 μm voxel size, 55 kVp, 145 μA, and 300 ms integration time (VivaCT, Scanco Medical AG, Switzerland). The region of interest was defined as 300 slices (6.3 mm) centered at the fracture midpoint and encompassing the whole callus. A threshold of 150/1 000 was set for all mineralized tissue, whereas a threshold of 460/1 000 was set for the CB only. Callus volume and callus BMD were calculated as previously described.^49^ After CT scanning, the bones were decalcified, embedded in paraffin, and sectioned at 6 μm thickness. For quantification of the cartilage callus, images of the H&E-stained sections were captured with Nanozoomer (Hamamatsu Photonics) and the cartilage area was measured with the NDP software.

### Statistics

Quantitative data were evaluated by Student’s *t* test or one-way ANOVA as indicated in figure legends. *P* < 0.05 was considered statistically significant.
